# The associations between maternal BMI and gestational weight gain and health outcomes in offspring at age 1 and 7 years

**DOI:** 10.1038/s41598-021-99869-7

**Published:** 2021-10-21

**Authors:** Valentina Chiavaroli, Sarah A. Hopkins, Janene B. Biggs, Raquel O. Rodrigues, Sumudu N. Seneviratne, James C. Baldi, Lesley M. E. McCowan, Wayne S. Cutfield, Paul L. Hofman, José G. B. Derraik

**Affiliations:** 1 Liggins Institute, University of Auckland, Auckland, New Zealand; 2 Neonatal Intensive Care Unit, Pescara Public Hospital, Pescara, Italy; 3 Department of Psychological Medicine, Faculty of Medical and Health Sciences, University of Auckland, Auckland, New Zealand; 4 Department of Health, Behavior and Society, College of Public Health, University of Kentucky, Lexington, KY USA; 5 Department of Paediatrics, Faculty of Medicine, University of Colombo, Colombo, Sri Lanka; 6 Department of Medicine, Dunedin School of Medicine, University of Otago, Dunedin, New Zealand; 7 Department of Obstetrics and Gynaecology, Faculty of Medical and Health Sciences, University of Auckland, Auckland, New Zealand; 8 A Better Start–National Science Challenge, University of Auckland, Auckland, New Zealand; 9 Department of Women’s and Children’s Health, Uppsala University, Uppsala, Sweden; 10 Research Institute for Health Sciences, Chiang Mai University, Chiang Mai, Thailand

**Keywords:** Endocrinology, Medical research

## Abstract

In secondary analyses of a randomised controlled trial of exercise during pregnancy, we examined associations between mid-pregnancy maternal body mass index (BMI) and excessive gestational weight gain (GWG) with offspring health. Follow-up data were available on 57 mother–child pairs at 1-year and 52 pairs at 7-year follow-ups. Clinical assessments included body composition and fasting blood tests. At age 1 year, increased maternal BMI in mid-gestation was associated with greater weight standard deviation scores (SDS) in the offspring (p = 0.035), with no observed associations for excessive GWG. At age 7 years, greater maternal BMI was associated with increased weight SDS (p < 0.001), BMI SDS (p = 0.005), and total body fat percentage (p = 0.037) in their children. Irrespective of maternal BMI, children born to mothers with excessive GWG had greater abdominal adiposity (p = 0.043) and less favourable lipid profile (lower HDL-C and higher triglycerides). At 7 years, maternal BMI and excessive GWG had compounded adverse associations with offspring adiposity. Compared to offspring of mothers with overweight/obesity plus excessive GWG, children of normal-weight mothers with adequate and excessive GWG were 0.97 and 0.64 SDS lighter (p = 0.002 and p = 0.014, respectively), and 0.98 and 0.63 SDS leaner (p = 0.001 and p = 0.014, respectively). Both greater maternal BMI in mid-pregnancy and excessive GWG were independently associated with increased adiposity in offspring at 7 years.

## Introduction

Pregnancy with obesity is associated with adverse short- and long-term consequences to offspring health and wellbeing^1–4^. A systematic review and meta-analysis^5^ showed that the offspring of women with prepregnancy overweight/obesity had an increased risk of high birth weight, and overweight and obesity in childhood and adolescence. Similarly, a large epidemiological study on 26,561 pairs of mothers and first-born daughters in Sweden showed that the odds of obesity in adulthood were 3.94 times higher in daughters born to mothers with obesity^1^. In addition, increasing maternal prepregnancy body mass index (BMI) was associated with adverse metabolic outcomes in offspring, such as reduced insulin sensitivity and increased blood pressure^6,7^.

Excessive gestational weight gain (GWG) is another important early-life risk factor for higher neonatal and childhood adiposity^8–10^. Offspring health risks associated with excessive GWG include (but are not limited to) increased adiposity and higher blood pressure at birth, in childhood, and adulthood^8,11,12^. Thus, limiting excessive GWG is recommended for the health of both mother and offspring^13,14^. The Institute of Medicine (IOM) guidelines define optimal ranges of maternal weight gain during pregnancy^15^. However, only 30–55% of pregnant women meet these guidelines, with a substantial proportion exceeding their recommended GWG^8,11,16,17^.

In 2016, Jin et al*.* found that maternal prepregnancy overweight/obesity and excessive GWG were associated with rapid offspring growth from birth to 3 years old^18^. A recent meta-analysis used data on 162,129 mothers and their children from 37 pregnancy and birth cohort studies from Europe, North America, and Australia^19^. It showed that higher maternal prepregnancy BMI and GWG were associated with an increased risk of childhood overweight/obesity, with the strongest effects in late childhood^19^. Still, it would be relevant to examine further the independent and combined effects of maternal prepregnancy BMI and excessive GWG on offspring adiposity and other relevant metabolic health outcomes. In a randomised controlled trial, we showed that moderate-intensity exercise in healthy nulliparous women over the second half of pregnancy led to an average birth weight reduction of ~ 250 g, but without differences in GWG^20^. These offspring were followed up until approximately 7 years of age^21^. Here, we examined whether maternal BMI at 20 weeks of gestation and/or excessive GWG were associated with alterations in body composition and metabolism in the offspring at birth and at approximately 1 and 7 years of age.

## Methods

### Ethics approval

Ethics approval for the original study was provided by the Multiregion Ethics Committee (Ministry of Health, New Zealand; AKX/04/08/227). Written informed consent was obtained from parents or guardians, and verbal or written consent from each child as appropriate to their age. This study was performed following all applicable institutional and international guidelines and regulations for medical research, in line with the principles of the Declaration of Helsinki.

### Participants

This study involved the follow-up of mothers and their offspring who participated in a community-based randomised controlled trial of exercise in pregnancy^20^. The original study included 84 healthy nulliparous women with a singleton pregnancy of fewer than 24 weeks of gestation who were relatively sedentary. Participants were randomly assigned to exercise (n = 47) or control (n = 37) groups.

At trial entry, maternal height and weight were measured, and BMI was calculated. GWG was calculated as the average weight gain per week during the second and third trimesters. Adequate GWG was defined as per IOM guidelines^15^: for underweight mothers (BMI < 18.5 kg/m^2^) 0.44–0.58 kg/week; normal-weight mothers (BMI 18.5–24.99 kg/m^2^) 0.35–0.50 kg/week; mothers with overweight (BMI 25.0–29.99 kg/m^2^) 0.23–0.33 kg/week; and mothers with obesity (BMI ≥ 30.0 kg/m^2^) 0.17–0.27 kg/week. Maternal and paternal heights were recorded during the trial, with mid-parental height standard deviation scores (MPHSDS) calculated^22^.

### Neonatal period

A study investigator measured neonatal anthropometry within 48 h of birth, namely birth weight to the nearest 10 g using electronic infant scales and crown-heel length using a neonatometer, with ponderal index subsequently calculated (in g/cm^3^). Birth weight and length were transformed into standard deviation scores (SDS, adjusted for age and sex) as per Niklasson et al.^23^. Offspring body composition (total body fat percentage) was assessed at 2 to 3 weeks postpartum by whole-body dual-energy x-ray absorptiometry (DXA) scans (Lunar Prodigy BX-1L; Lunar Corp., Madison, WI, USA).

### 1-year assessment

Children underwent anthropometric assessments at approximately 1 year of age, including weight, length, abdominal circumference, and hip circumference, as previously described^21^, with their BMI subsequently calculated. Length, weight, and BMI were transformed into SDS^24^, with height also adjusted for their genetic potential (parents’ heights): child’s height SDS minus MPHSDS.

### 7-year assessment

Clinical examinations in the children included anthropometric measurements and pubertal staging, as previously described^21^, and all participants were prepubertal corresponding to Tanner stage 1. Briefly, height was measured to the nearest mm using a Harpenden stadiometer, and weight using calibrated electronic scales in light clothing to the nearest 10 g. Height, weight, and BMI were transformed into age- and sex-adjusted SDS^25^. Overweight was defined as a BMI SDS ≥ 1.036 but < 1.645 (85th–94th percentile for age and sex), and obesity as BMI SDS ≥ 1.645 (≥ 95th percentile)^26^. Body composition was assessed using DXA scans (DXA Lunar Prodigy 2000; General Electric, Madison, WI, USA). Key parameters of interest were total body fat percentage and the android-to-gynoid-fat ratio (an indicator of abdominal adiposity).

After a five-minute rest and with the child at a sitting position, systolic and diastolic blood pressures were measured on the non-dominant arm, with an appropriately-sized cuff using the same calibrated sphygmomanometer (Dinamap ProCare 100; GE Healthcare, Chalfont St Giles, UK).

Blood samples were obtained after an overnight fast to measure serum glucose, insulin, total cholesterol, high-density lipoprotein cholesterol (HDL-C), low-density lipoprotein cholesterol (LDL-C), and triglycerides. The homeostasis model assessment of insulin resistance (HOMA-IR) was used as a surrogate marker of insulin sensitivity^27^.

Food diaries were also collected, with nutritional intake estimated using standard household measures and/or food labels. Records were entered into Foodworks software (v5.0, Xyris Software, Brisbane, Australia) by a trained investigator, as previously described^21^. Children’s physical activity levels were estimated using a brief questionnaire.

### Assays

Insulin concentrations were measured by electrochemiluminescence immunoassay using a COBAS e411 autoanalyser (Hitachi High Technologies Corporation, Tokyo, Japan), with an inter-assay coefficient of variation (CV) of 2.4%. Glucose, triglycerides, total cholesterol, and HDL-C concentrations were measured by enzymatic colorimetric assay (Roche, Mannheim, Germany) on a COBAS c311 autoanalyser (Hitachi), with inter-assay CVs of 2.0, 1.4, 2.8, and 1.9%, respectively.

### Statistical analyses

Demographic and lifestyle characteristics between groups were compared with one-way ANOVA (continuous variables) and Chi-square tests (categorical variables).

Neonatal outcomes were analysed using general linear models. Models included maternal BMI (as a continuous variable) and GWG group (excessive or not) as key predictors, and also adjusted for child’s sex, gestational age at birth, and group allocation in the original trial, with MPHSDS also added as a covariate for length SDS. Outcomes at the 1-year and 7-year follow-ups were similarly examined, except that gestational age was replaced by the child’s age at the assessment.

Potential interactions between maternal BMI, GWG group, and sex were examined in statistical models but subsequently removed if not statistically significant. Where a significant interaction was present, the results were reported separately as appropriate. In addition, exploratory analyses were run including birth weight SDS as a mediator, or the child’s reported levels of exercise or total energy intake as a covariate.

Associations with maternal BMI are reported as the adjusted β coefficients and respective 95% confidence intervals (CI). Data for GWG groups are reported as the least-squares means (i.e., model-adjusted means) and 95% CI, as well as the adjusted mean differences (aMD) with respective 95% CI (when relevant).

The likelihood of overweight at the 7-year follow-up was assessed using a generalised linear model, including the same predictors as for continuous outcomes, with the result reported as the adjusted relative risk and 95% CI.

Lastly, the cohort was stratified into four groups according to a combination of maternal BMI and GWG:

(1) normal weight & adequate GWG; (2) normal weight & excessive GWG; (3) overweight/obesity & adequate GWG; and (4) overweight/obesity & excessive GWG. Key outcomes at the 7-year follow-up (i.e., weight SDS, BMI SDS, and total body fat percentage) were compared between groups using general linear regression models constructed as described earlier for the stratified analyses, except for the removal of maternal BMI as a covariate, with pairwise comparisons between groups reported.

Outcomes were examined for normality and, if necessary, were log-transformed to approximate a normal distribution and subsequently reported as back-transformed values. Statistical analyses were performed in SAS v9.4 (SAS Institute, Cary, USA) and SPSS v25 (IBM Corp, Armonk, NY, USA). All tests were two-tailed, with statistical significance maintained at p < 0.05, without adjustment for multiple comparisons as per Rothman^28^. There was no imputation of missing values.

## Results

Of the initial 84 women and their children who participated in the original trial, 1-year follow-up data were available on 57 children (37 from mothers in the exercising group and 20 from control mothers). At trial entry, 28 mothers (49%) had overweight or obesity, and at the end of pregnancy, 39 mothers (68%) had excessive GWG as per IOM guidelines. Data at the 7-year follow-up were available on 52 children (31 from exercising mothers, and 21 from control mothers); at study entry, these mothers were at a mean gestational age of 19.5 weeks (SD = 1.1; range 17.0–23.4 weeks), and 25 (48%) had overweight or obesity. At the end of pregnancy, there were 35 (67%) mothers with excessive GWG. Maternal parameters at study entry were similar in the follow-up and lost-to-follow-up groups, as were most offspring parameters at birth (Supplementary Table 1).

### Neonatal outcomes

Every 1.0 kg/m^2^ increase in maternal BMI at trial recruitment was associated with a 0.056 SDS increase in offspring birth weight (95% CI 0.002, 0.111; p = 0.044; Table 1), but the latter was not different between the offspring of mothers who had excessive or adequate GWG (Table 2). Neither maternal BMI nor GWG groups were associated with other neonatal outcomes assessed (Tables 1 and 2).

**Table 1:** Neonatal anthropometry and body composition measured in the offspring (n = 77) in association with maternal body mass index (BMI) in mid-gestation.

	β (95% CI)	*p*-value
Birth weight SDS	0.056 (0.002, 0.111)	**0.044**
Birth length SDS	0.026 (− 0.039, 0.091)	0.43
Ponderal index (g/cm^3^)	0.006 (− 0.009, 0.021)	0.44
Total body fat (%)	− 0.007 (− 0.180, 0.165)	0.93

Data are β coefficients and 95% CI derived from general linear models, including maternal gestational weight gain, and adjusted for child’s sex, gestational age at birth, and trial group allocation; mid-parental height SDS was also added as a covariate for length SDS.

Body composition (total body fat) was measured at 2 to 3 weeks postpartum.

CI, confidence interval; SDS, standard deviation score.

*p*-values for statistically significant associations (at p < 0.05) are shown in bold.

**Table 2:** Neonatal anthropometry and body composition measured in the offspring according to maternal gestational weight gain (GWG).

	Excessive GWG	Adequate GWG	*p*-value
n	53	24	
Birth weight SDS	0.13 (− 0.11, 0.36)	− 0.15 (− 0.50, 0.20)	0.20
Birth length SDS	0.40 (0.11, 0.68)	0.44 (0.02, 0.85)	0.43
Ponderal index (g/cm^3^)	2.65 (2.59, 2.72)	2.63 (2.54, 2.73)	0.76
Total body fat (%)	8.3 (7.6, 9.0)	8.5 (7.3, 9.6)	0.82

Data are model-adjusted means and the respective 95% confidence intervals (CI) derived from general linear models, including maternal body mass index (BMI) in mid-gestation, and adjusted for child’s sex, gestational age at birth, and trial group allocation; mid-parental height SDS was also added as a covariate for length SDS.

Body composition (total body fat) was measured at 2 to 3 weeks postpartum.

SDS, standard deviation score.

### 1-year follow-up

Offspring were assessed at a median age of 1.1 years (range 1.0–1.3 years) (Supplementary Table 2). Increased maternal BMI was associated with greater weight SDS (+ 0.09 SDS per + 1 kg/m^2^ of maternal BMI; p = 0.035), with a similar trend for BMI SDS (+ 0.09 SDS per + 1 kg/m^2^ of maternal BMI; p = 0.058) (Table 3). However, maternal BMI was not associated with other anthropometric measurements (Table 3). There were no differences in anthropometry in the offspring of mothers with excessive GWG compared to adequate GWG when maternal BMI was accounted for (Table 4).

**Table 3:** Anthropometry at the 1-year follow-up in the offspring (n = 57) in association with maternal body mass index (BMI) in mid-gestation.

	β (95% CI)	*p*-value
Weight SDS	0.090 (0.007, 0.173)	**0.035**
Height SDS–MPHSDS	0.048 (− 0.032, 0.128)	0.23
BMI SDS	0.086 (− 0.003, 0.175)	0.058
Abdominal circumference (cm)	0.15 (− 0.11, 0.41)	0.25
Hip circumference (cm)	0.23 (− 0.06, 0.53)	0.12

Data are β coefficients and 95% confidence intervals (CI) derived from general linear models, including maternal gestational weight gain, adjusted for child’s sex, age, and trial group allocation.

*p*-values for statistically significant associations (at p < 0.05) are shown in bold.

MPHSDS, mid-parental height standard deviation score; SDS, standard deviation score.

**Table 4:** Anthropometry at the 1-year follow-up in the offspring according to maternal gestational weight gain (GWG).

	Excessive GWG	Adequate GWG	*p*-value
n	39	18	
Weight SDS	− 0.11 (− 0.44, 0.22)	− 0.44 (− 0.91, 0.04)	0.25
Height SDS–MPHSDS	− 0.16 (− 0.48, 0.16)	− 0.12 (− 0.57, 0.33)	0.89
BMI SDS	− 0.34 (− 0.7, 0.01)	− 0.44 (− 0.95, 0.07)	0.75
Abdominal circumference (cm)	46.6 (45.5, 47.6)	46.7 (45.2, 48.2)	0.89
Hip circumference (cm)	42.8 (41.7, 43.8)	44.1 (42.4, 45.7)	0.20

Data are model-adjusted means and the respective 95% confidence intervals (CI) derived from general linear models, including maternal body mass index (BMI) in mid-gestation, and adjusted for child’s sex, age, and trial group allocation.

MPHSDS, mid-parental height standard deviation score; SDS, standard deviation score.

### 7-year follow-up

At this second follow-up, children were re-assessed at a median age of 7.7 years (range 6.1–8.9 years) (Supplementary Table 2). Maternal BMI in mid-gestation was associated with markers of adiposity in the offspring, with every additional 1 kg/m^2^ associated with increases of 0.10 SDS in weight (p < 0.001), 0.084 SDS in BMI (p = 0.005), with total body fat percentage also becoming 2.4% greater (p = 0.037) with a consequent reduction in lean mass (Table 5). In addition, the mothers of the seven children with overweight at the 7-year assessment (there were none with obesity) had a markedly greater BMI at baseline compared with the mothers of the 45 normal-weight children (29.6 ± 4.8 kg/m^2^ vs 24.1 ± 2.6 kg/m^2^; p < 0.0001).

**Table 5:** Clinical outcomes at the 7-year follow-up in the offspring (n = 52) in association with maternal body mass index (BMI) in mid-gestation.

		β (95% CI)	*p*-value
Anthropometry	Weight SDS	0.102 (0.046, 0.159)	** < 0.001**
	Height SDS–MPHSDS	0.078 (− 0.005, 0.160)	0.06
	BMI SDS	0.084 (0.027, 0.142)	**0.005**
Body composition	Total body fat (%)^a^	1.024 (1.002, 1.046)	**0.037**
	Lean mass (%)	− 0.40 (− 0.76, − 0.03)	**0.035**
	Android-to-gynoid-fat ratio	0.002 (− 0.009, 0.013)	0.72
Blood pressure	Systolic (mmHg)	− 0.12 (− 1.04, 0.81)	0.80
	Diastolic (mmHg)	− 0.28 (− 0.97, 0.41)	0.42
Glucose homeostasis	Fasting glucose (mmol/L)	− 0.02 (− 0.06, 0.02)	0.37
	Fasting insulin (mIU/L)^a^	1.032 (0.970, 1.094)	0.28
	HOMA-IR^a^	1.028 (0.962, 1.098)	0.40
Lipid profile	Total cholesterol (mmol/L)	− 0.02 (− 0.09, 0.05)	0.59
	HDL-C (mmol/L)	0.00 (− 0.02, 0.02)	0.91
	LDL-C (mmol/L)	− 0.02 (− 0.09, 0.065)	0.60
	Triglycerides (mmol/L)	0.00 (− 0.02, 0.02)	0.74
	Total cholesterol/HDL-C	− 0.01 (− 0.08, 0.05)	0.66
	Triglycerides/HDL-C	0.00 (− 0.02, 0.02)	0.78

Data are β coefficients and 95% confidence intervals (CI) derived from general linear models, including maternal body mass index (BMI) in mid-gestation, adjusted for child’s sex, age, and trial group allocation.

HDL-C, high-density lipoprotein cholesterol; HOMA-IR, homeostatic model assessment of insulin resistance; LDL-C, low-density lipoprotein cholesterol; MPHSDS, mid-parental height standard deviation score; SDS, standard deviation score.

*p*-values for statistically significant associations (at p < 0.05) are shown in bold.

^a^Back-transformed β coefficients represent the proportional change in the outcome in association with maternal BMI.

Independent of maternal BMI, children born to mothers with excessive GWG had greater abdominal adiposity than those born to mothers with adequate GWG (android-to-gynoid-fat ratio: 0.64 vs 0.55; p = 0.043) (Table 6). No children born to mothers with adequate GWG (n = 17) had overweight compared to 20% (7/35) of those born to mothers with excessive GWG, with the adjusted relative risk of overweight in the latter group nearly eight times greater [7.8 (95% CI 1.3, 48.5); p = 0.027].

Notably, for weight SDS there was an interaction between GWG group and sex (p = 0.020), where boys born to mothers with excessive GWG were 0.77 SDS heavier (aMD 95% CI 0.22, 1.33; p = 0.007) than boys of mothers with adequate GWG [0.72 (95% CI 0.39, 104) vs − 0.06 (95% CI − 0.51, 0.40)]. In contrast, there were no differences among girls in the two groups [0.23 (95% CI − 0.03, 0.48) vs 0.47 (95% CI − 0.01, 0.96), respectively; aMD − 0.24 (95% CI − 0.84, 0.36); p = 0.43].

In addition, children born to mothers with excessive GWG had a less favourable lipid profile, with concentrations of HDL-C 10.3% lower (p = 0.038), triglyceride 38.1% higher (p = 0.003), total cholesterol to HDL-C ratio 22.7% higher (p = 0.024), and triglycerides to HDL-C ratio 61.1% higher (p = 0.003) (Table 6). However, there were no observed differences in blood pressure or glucose homeostasis (Table 6). Importantly, there were no differences in macronutrient intake or physical activity levels between GWG groups (Supplementary Table 3).

**Table 6:** Clinical outcomes at the 7-year follow-up in the offspring according to maternal gestational weight gain.

		Excessive GWG	Adequate GWG	*p*-value
n		35	17	
Anthropometry	Weight SDS	0.48 (0.24, 0.72)	0.17 (− 0.17, 0.52)	0.14
	Height SDS–MPHSDS	0.12 (− 0.23, 0.47)	0.26 (− 0.21, 0.74)	0.63
	BMI SDS	0.22 (− 0.03, 0.46)	− 0.17 (− 0.51, 0.18)	0.07
Body composition	Total body fat (%)	16.6 (15.2, 18.1)	15.4 (13.5, 17.6)	0.37
	Lean mass (%)	78.0 (76.5, 79.5)	79.4 (77.1, 81.6)	0.30
	Android-to-gynoid-fat ratio	0.64 (0.59, 0.68)	0.55 (0.49, 0.62)	**0.043**
Blood pressure	Systolic (mmHg)	98.7 (94.9, 102.5)	97.5 (91.5, 103.4)	0.72
	Diastolic (mmHg)	58.8 (56.0, 61.6)	56.7 (52.2, 61.1)	0.42
Glucose homeostasis	Fasting glucose (mmol/L)	4.76 (4.59, 4.94)	4.95 (4.69, 5.22)	0.23
	Fasting insulin (mIU/L)	6.02 (4.72, 7.67)	6.11 (4.23, 8.84)	0.94
	HOMA-IR	1.27 (0.97, 1.67)	1.34 (0.89, 2.03)	0.83
Lipid profile	Total cholesterol (mmol/L)	4.81 (4.51, 5.12)	4.46 (4.00, 4.92)	0.201
	HDL-C (mmol/L)	1.57 (1.48, 1.66)	1.75 (1.61, 1.89)	**0.038**
	LDL-C (mmol/L)	3.06 (2.75, 3.36)	2.58 (2.11, 3.04)	0.09
	Triglycerides (mmol/L)	0.87 (0.79, 0.96)	0.63 (0.50, 0.76)	**0.003**
	Total cholesterol/HDL-C	3.13 (2.86, 3.39)	2.55 (2.14, 2.96)	**0.024**
	Triglycerides/HDL-C	0.58 (0.50, 0.65)	0.36 (0.24, 0.47)	**0.003**

Data are model-adjusted means and the respective 95% confidence intervals (CI) derived from general linear models, including maternal body mass index (BMI) in mid-gestation, adjusted for child’s sex, age, and trial group allocation.

HDL-C, high-density lipoprotein cholesterol; HOMA-IR, homeostatic model assessment of insulin resistance; LDL-C, low-density lipoprotein cholesterol; MPHSDS, mid-parental height standard deviation score; SDS, standard deviation score.

*p*-values for statistically significant differences between the two groups (at p < 0.05) are shown in bold.

Mediation analyses run with the inclusion of birth weight SDS as a covariate did not alter the results (data not shown). While data on the children’s reported levels of exercise or energy intake were not available for all participants (Supplementary Table 3), exploratory models including these parameters as covariates corroborated the overall findings on the associations between maternal BMI and GWG and offspring outcomes (data not shown).

### High maternal BMI and excessive GWG

Study outcomes were also examined in the offspring of mothers stratified according to a combination of mid-pregnancy BMI and GWG (Table 7). At the 7-year follow-up, there was evidence of a progressive increase in weight and BMI according to their mother’s weight and GWG (Table 7). Compared to the offspring born to mothers with overweight/obesity as well as excessive GWG, children of normal-weight mothers with adequate and excessive GWG were 0.97 and 0.64 SDS lighter (p = 0.002 and p = 0.014, respectively), and 0.98 and 0.63 SDS leaner (p = 0.001 and p = 0.014, respectively) (Table 7). Of note, all seven children who were found to be overweight at 7 years were born to mothers with both overweight/obesity and excessive GWG (Table 7).

**Table 7:** Demographic and anthropometric outcomes at the 7-year follow-up in the offspring born to mothers stratified according to maternal body mass index (BMI) in mid-gestation and maternal gestational weight gain (GWG).

		Normal weight Adequate GWG	Normal weight Excessive GWG	Overweight/obesity Adequate GWG	Overweight/obesity Excessive GWG
n		10	17	7	18
Mothers	BMI at baseline (kg/m^2^)	22.0 ± 1.7	22.6 ± 1.5	27.0 ± 1.7	27.6 ± 3.5
	GWG (kg/week)	0.36 ± 0.09	0.62 ± 0.11	0.27 ± 0.05	0.61 ± 0.14
	Age (years)	29.3 ± 2.7	30.5 ± 4.0	31.0 ± 2.1	32.3 ± 3.3
	Ethnicity (NZE)	6 (60%)	14 (82%)	6 (86%)	15 (83%)
Children	Sex (females)	9 (64%)	11 (44%)	4 (40%)	12 (43%)
	Weight SDS	− 0.14 (− 0.63, 0.35)**	0.19 (− 0.16, 0.54)*	0.43 (− 0.14, 1.00)	0.83 (0.48, 1.19)
	BMI SDS	− 0.42 (− 0.90, 0.05)**	− 0.07 (− 0.42, 0.27)*	0.05 (− 0.51, 0.61)	0.56 (0.21, 0.90)
	Total body fat (%)	15.8 (12.1, 19.5)	16.2 (13.7, 18.6)	16.2 (12.3, 20.0)	18.9 (16.5, 21.3)
	Overweight	Nil	Nil	Nil	7 (39%)

Categorical data are n (%); continuous maternal data are means ± standard deviations; continuous data for the children are model-adjusted means and respective 95% confidence intervals derived from general linear models, adjusted for sex, age, and trial group allocation.

NZE, New Zealand European; SDS, standard deviation score.

*p < 0.05 and **p < 0.01 for pairwise differences in comparison to mothers with both overweight/obesity and excessive GWG.

## Discussion

Greater maternal BMI in mid-pregnancy was associated with increased adiposity in the offspring at approximately 7 years of age. Irrespective of maternal BMI in mid-pregnancy, excessive GWG was also associated with adverse metabolic health outcomes in the offspring (e.g. less favourable lipid profile). However, these adverse outcomes were magnified in the offspring of mothers with both high maternal BMI and excessive GWG. Thus, our comprehensive longitudinal assessments of cardio-metabolic parameters provide additional evidence on the independent and combined potential effects of maternal mid-pregnancy BMI and excessive GWG on offspring health.

A higher maternal BMI has been shown to have an adverse effect on offspring cardio-metabolic profile, both in the short and long terms^5,7,10^. Greater adiposity and unfavourable metabolic outcomes (e.g., insulin resistance, diabetes, and higher blood pressure) are among the many adverse effects observed in the offspring of women with overweight or obesity^5,7^. Indeed, in a cohort analysis involving 37,709 adult offspring in the UK, offspring born to mothers with obesity had an increased risk of premature all-cause death (hazard ratio 1.35; 95% CI 1.17, 1.55) and hospital admission for a cardiovascular event (hazard ratio 1.29; 95% CI 1.06, 1.57) compared with those born to normal-weight mothers^29^. GWG also has relevant implications for newborns (e.g. macrosomia)^30^ and for the long-term health of offspring (e.g. obesity and higher blood pressure)^11,12,31^. Thus, our findings are of concern as they corroborate findings from previous studies, being particularly relevant in light of the global obesity epidemic^32–34^.

Numerous approaches have been developed to improve maternal weight status before conception^35^ and during pregnancy^36–38^, and thus reduce the likelihood of adverse effects on offspring health and wellbeing. Two systematic reviews and meta-analyses have shown that physical activity and dietary interventions may limit maternal weight gain during pregnancy, lowering the risk of adverse fetal outcomes^36,37^. In our previous randomised controlled trial of exercise in pregnancy, we observed an average birth weight reduction of approximately 250 g in the offspring of mothers in the exercising group, without differences in GWG between groups^20^. Notably, the 7-year follow-up of these offspring showed that initiation of exercise during pregnancy amongst relatively sedentary mothers might lead to increased adiposity in childhood^21^. However, there was no reduction in maternal BMI in our trial^20^; thus, exercise initiated during pregnancy among women with overweight/obesity may only benefit the offspring if there is an associated reduction in maternal BMI and maintenance of adequate GWG. Ultimately, the optimal strategy would be addressing the obesity issue long before pregnancy to optimise the in utero environment for the offspring.

Follow-up studies assessing the implications of being born to mothers with excessive adiposity over time are needed. This is particularly true given that the alterations in offspring body composition and metabolism may be expressed and amplified later in life^5,7,11,12,31^. At our 1-year follow-up, there were no observed differences in anthropometry between children born to women with normal weight or overweight/obesity in mid-pregnancy, as was the case for the offspring of children born to women with excessive or adequate GWG. However, when follow-up was extended into childhood, we found that greater maternal BMI in mid-pregnancy was associated with increased adiposity in the offspring at 7 years. This corroborates the existing literature linking greater offspring adiposity and risk of obesity with increasing maternal prepregnancy BMI^1,39–41^. In a recent analysis involving 27,016 maternal-child dyadic records, a 4.5% increase in the risk of offspring obesity at a mean age of 1.7 years was observed for each 1 kg/m^2^ increase in prepregnancy BMI compared with children born to normal-weight mothers^42^. In the same study, children whose mothers had excessive GWG were 50% more likely to have obesity than those whose mothers had adequate GWG^42^. Similarly, at the 7-year assessment of this study, we found that a fifth of children born to mothers with excessive GWG had overweight/obesity compared with none of those born to mothers with adequate GWG. Moreover, independent of maternal BMI, children born to mothers with excessive GWG had greater abdominal adiposity than those born to mothers with adequate GWG, consistent with previous studies^8–10,43^.

Notably, we also found that children born to mothers with excessive GWG had a less favourable lipid profile, despite the absence of differences in macronutrient intake or physical activity levels. At the 9-year follow-up of a population-based birth cohort study in the UK involving 3457 mother–offspring pairs (for blood assays), women with excessive GWG were more likely to have offspring with lower HDL-C and apolipoprotein A1 levels^44^. In another cohort study involving 12,775 children aged 2–9 years recruited from eight European countries, total cholesterol and LDL-C differed across GWG tertiles but without differences in HDL-C and triglycerides^45^. While further studies are needed, the current evidence indicates that overnutrition in utero adversely impacts the offspring’s cardio-metabolic profile. Our findings suggest the unfavourable effects of an adverse intrauterine environment on offspring health may become evident over time, highlighting the importance of a longer follow-up into adolescence, when adiposity amplifies and the associations with long-term health risk become clearer^46,47^.

The mechanisms underlying the effects of maternal adiposity on the next generation remain to be elucidated. Increasing rates of maternal obesity characterise the proposed “obesity cycle”, underpinning in utero programming of later adiposity and transgenerational amplification of obesity^48,49^. Overnutrition in utero has been associated with early postnatal obesity and other metabolic sequelae either directly or via epigenetic mechanisms^50,51^. Indeed, animal models of perinatal overnutrition showed long-lasting modifications in the structure and function of appetite-regulating centres in the brain^52^.

Our study's main limitation was the loss of participants (approximately 32% at the 1-year and 38% at the 7-year follow-up), which reduced our sample size and affected our statistical power. In addition, GWG in our study was calculated based on weight recorded in mid-pregnancy instead of the women’s prepregnancy weight. However, most pregnancies are unplanned, and most women do not seek pre-conceptional care^53^. Consequently, obtaining prepregnancy weight is challenging, and most studies rely on self-reported data. While the absence of prepregnancy data could have led to misclassification of GWG in some women, a large study on 1000 women using actual measurements reported that maternal weight and body composition were essentially unchanged throughout the first trimester of pregnancy^54^. Further, mothers in our study participated in an exercise intervention, although their group allocation was controlled for in the analyses. Lastly, the exploratory nature of some of our analyses needs to be considered. Thus, some caution is required when extrapolating our findings to all pregnant women and their children. Nevertheless, the strengths of our study are data on a comprehensive range of anthropometric, body composition, and metabolic outcomes, as well as estimates of dietary intake and physical activity levels.

In conclusion, greater maternal BMI in mid-pregnancy and excessive GWG exert a negative impact on offspring health. Importantly, excessive GWG is also associated with a less favourable lipid profile in childhood, irrespective of maternal BMI. Given the detrimental effects of increased maternal adiposity on offspring health and wellbeing, more work is needed to address the increasing number of women entering pregnancy with obesity. Studies incorporating a comprehensive range of outcomes (such as those reported here) should be repeated in a larger sample. Lastly, it is essential to identify effective preventative strategies to halt the worsening intergenerational cycle of obesity and associated comorbidities.

## Supplementary Information

Supplementary Information.

## Data Availability

The study data cannot be made available in a public repository due to the strict conditions of the ethics approval. However, the anonymised data used in this manuscript could be made available to other investigators upon bona fide request, following all the necessary approvals (including ethics) of the detailed study proposal and statistical analyses plan. Any queries should be directed to Prof Paul Hofman (p.hofman@auckland.ac.nz).
