# Supplementary material for: The associations between maternal BMI and gestational weight gain and health outcomes in offspring at age 1 and 7 years
Source: Sci Rep. 2021 Oct 21;11:20865. doi: 10.1038/s41598-021-99869-7 (PMC8531053; doi:10.1038/s41598-021-99869-7)
Supplement: Supplementary file 1 — Supplementary Information. [file 41598_2021_99869_MOESM1_ESM.pdf]

Supplementary File

The associations between maternal BMI and gestational weight gain and health outcomes in offspring at age 1 and 7 years

Valentina Chiavaroli[1,2], Sarah A Hopkins[1,3], Janene B Biggs[1], Raquel O Rodrigues[1,4], Sumudu N Seneviratne[1,5], James C Baldi[6], Lesley M E McCowan[7], Wayne S Cutfield[1,8], Paul L Hofman[1,*], José G B Derraik[1,9,10,*]

[1] Liggins Institute, University of Auckland, Auckland, New Zealand
[2] Neonatal Intensive Care Unit, Pescara Public Hospital, Pescara, Italy
[3] Department of Psychological Medicine, Faculty of Medical and Health Sciences, University of Auckland, Auckland, New Zealand
[4] Department of Health, Behavior and Society, College of Public Health, University of Kentucky, Lexington, KY, United States
[5] Department of Paediatrics, Faculty of Medicine, University of Colombo, Colombo, Sri Lanka
[6] Department of Medicine, Dunedin School of Medicine, University of Otago, Dunedin, New Zealand
[7] Department of Obstetrics and Gynaecology, Faculty of Medical and Health Sciences, University of Auckland, Auckland, New Zealand
[8] A Better Start – National Science Challenge, University of Auckland, Auckland, New Zealand
[9] Department of Women's and Children's Health, Uppsala University, Uppsala, Sweden
[10] Research Institute for Health Sciences, Chiang Mai University, Chiang Mai, Thailand

* Authors for correspondence:

Dr José Derraik – Liggins Institute, University of Auckland, Private Bag 92019, Auckland, New Zealand; E-mail: j.derraik@auckland.ac.nz

Prof Paul L Hofman – Liggins Institute, University of Auckland, Private Bag 92019, Auckland, New Zealand; E-mail: p.hofman@auckland.ac.nz

Supplementary Table S1

Maternal and offspring parameters in the follow-up and lost groups.

			FOLLOW-UP	LOST TO FOLLOW-UP	*P*-VALUE
**n**			57	27	
**Mother**	**Demography**	Age (years)	31.2 ± 3.4	31.2 ± 3.8	0.98
		Ethnicity (NZ European)	52 (91%)	26 (96%)	0.40
	**Anthropometry**	Early-pregnancy maternal BMI (kg/m^2)	25.1 ± 3.8	26.0 ± 3.7	0.33
		BMI status (overweight/obesity)	28 (50%)	14 (52%)	0.95
		Weight gain (kg/week)	0.52 ± 0.18	0.54 ± 0.19	0.77
		Excessive gestational weight gain	35 (67%)	18 (72%)	0.68
**Offspring**	**At birth**	Gestational age (days)	279 ± 10	279 ± 9	0.99
		Birth weight SDS	-0.11 ± 0.89	0.24 ± 0.75	0.09
		Sex (males)	31 (54%)	14 (52%)	0.83

BMI, body mass index; SDS, standard deviation score.
Data are provided as means ± standard deviations or n (%), as appropriate.

Supplementary Table S2

Demographic characteristics of the offspring at the 1-year and 7-year follow-ups, according to maternal gestational weight gain (GWG).

		OVERALL	ADEQUATE GWG	EXCESSIVE GWG	*P*-VALUE
**1-year follow-up**	**n**	57	18	39	
	**Age (years)**	1.1 [1.0, 1.1]	1.0 [1.0, 1.1]	1.1 [1.0, 1.1]	0.60
	**Sex (males)**	30 (53%)	10 (56%)	20 (51%)	0.78
	**Ethnicity (NZE)**	45 (79%)	14 (78%)	31 (80%)	0.99
**7-year follow-up**	**n**				
	**Age (years)**	7.7 [6.9, 8.3]	7.9 [6.9, 8.3]	7.6 [7.1, 8.3]	0.97
	**Sex (males)**	28 (54%)	9 (53%)	19 (54%)	0.99
	**Ethnicity (NZE)**	41 (79%)	12 (71%)	29 (83%)	0.47

Age data are medians [quartile 1, quartile 3]; sex and ethnicity data are n (%).
NZE, New Zealand European.

Supplementary Table S3

Dietary intake and physical activity at the 7-year follow-up among children born to mothers with excessive or adequate gestational weight gain (GWG), who completed the questionnaires on dietary intake and physical activity.

		ADEQUATE GWG	EXCESSIVE GWG	*P*-VALUE
**n**		11	27	
**Demography**	Age (years)	7.6 ± 0.9	7.6 ± 0.8	0.95
	Sex (males)	7 (64%)	16 (59%)	0.99
	Ethnicity (NZ European)	9 (82%)	23 (85%)	0.99
**Daily dietary intake**	Energy (kJ)	6861 ± 1196	6864 ± 1342	0.99
	Fat (g)	55 ± 20	55 ± 12	0.97
	Sugar (g)	94 ± 26	88 ± 35	0.58
	Dietary fibre (g)	19 ± 9	22 ± 6	0.22
	Total energy from fat (%)	29 ± 7	30 ± 7	0.73
	Total energy from protein (%)	16 ± 4	17 ± 4	0.45
	Total energy from carbohydrate (%)	53 ± 5	51 ± 9	0.45
**Physical activity**	Time being physically active (hours/day)	3.0 [2.5, 3.0]	2.8 [2.5, 3.1]	0.99

Physical activity data are median [quartile 1, quartile 3]; other continuous data are means ± standard deviations; sex and ethnicity data are n (%).
